# Multi-epitope chimeric vaccine design against emerging Monkeypox virus *via* reverse vaccinology techniques- a bioinformatics and immunoinformatics approach

**DOI:** 10.3389/fimmu.2022.985450

**Published:** 2022-08-25

**Authors:** Sara Aiman, Yahya Alhamhoom, Fawad Ali, Noor Rahman, Luca Rastrelli, Asifullah Khan, Qurat ul Ain Farooq, Abbas Ahmed, Asif Khan, Chunhua Li

**Affiliations:** ^1^ Faculty of Environmental and Life Sciences, Beijing University of Technology, Beijing, China; ^2^ Department of Pharmaceutics, College of Pharmacy, King Khalid University, Abha, Saudi Arabia; ^3^ Department of Biochemistry, Hazara University, Mansehra, Pakistan; ^4^ Department of Biochemistry, Abdul Wali Khan University Mardan, Mardan, KP, Pakistan; ^5^ Dipartimento di Farmacia, University of Salerno, Via Giovanni Paolo II, Fisciano, SA, Italy; ^6^ Department of Biotechnology, Abdul Wali Khan University Mardan, Mardan, Pakistan; ^7^ Education department, Qurtaba University of Science and Information Technology (QUSIT) Peshawar, Peshawar, Pakistan

**Keywords:** reverse vaccinology, monkeypox virus, multi-epitope vaccine construct, vaccine candidates, immune simulation, molecular dynamic simulation

## Abstract

The emerging monkeypox virus (MPXV) is a zoonotic orthopoxvirus that causes infections in humans similar to smallpox. Since May 2022, cases of monkeypox (MPX) have been increasingly reported by the World Health Organization (WHO) worldwide. Currently, there are no clinically validated treatments for MPX infections. In this study, an immunoinformatics approach was used to identify potential vaccine targets against MPXV. A total of 190 MPXV-2022 proteins were retrieved from the ViPR database and subjected to various analyses including antigenicity, allergenicity, toxicity, solubility, IFN-γ, and virulence. Three outer membrane and extracellular proteins were selected based on their respective parameters to predict B-cell and T-cell epitopes. The epitopes are conserved among different strains of MPXV and the population coverage is 100% worldwide, which will provide broader protection against various strains of the virus globally. Nine overlapping MHC-I, MHC-II, and B-cell epitopes were selected to design multi-epitope vaccine constructs linked with suitable linkers in combination with different adjuvants to enhance the immune responses of the vaccine constructs. Molecular modeling and structural validation ensured high-quality 3D structures of vaccine constructs. Based on various immunological and physiochemical properties and docking scores, MPXV-V2 was selected for further investigation. *In silico* cloning revealed a high level of gene expression for the MPXV-V2 vaccine within the bacterial expression system. Immune and MD simulations confirmed the molecular stability of the MPXV-V2 construct, with high immune responses within the host cell. These results may aid in the development of experimental vaccines against MPXV with increased potency and improved safety.

## Introduction

The monkeypox virus (MPXV) is an orthopoxvirus belonging to the Poxviridae family that causes diseases in humans and animals. Monkeypox (MPX) is a zoonotic disease in which the virus is usually transmitted through animal-human contact, with symptoms similar to smallpox but with reduced mortality (1). MPXV is rather large (200-250 nanometers), brick-shaped with a lipoprotein envelope, and a linear double-stranded DNA genome (2). Currently, MPXVs are classified into two clades. The Central African Congo Basin clade has been reported more frequently than the West African clade. The Congo Basin clade has recorded occurrences of human-to-human transmission, whereas the West African clade has not (3). In May 2022, MPXV cases were reported by the World Health Organization (WHO). Several cases of MPX have been identified in geographically diverse countries. Human-to-human transmission of MPXV occurs because of close contact with lesions, bodily fluids, respiratory droplets, and infected objects such as bedding. Consumption of undercooked meat and other diseased animal products is a potential risk factor (4). The early symptoms of MPX include fatigue, headache, fever, myalgia, and lymphadenopathy- a key feature that differentiates MPX from smallpox. After 1-2 days, mucosal lesions appear in the mouth followed by centrifugally concentrated skin lesions on the face, hands, and feet. The rash can spread to the rest of the body and the number of lesions can vary from a few to thousands. MPX usually takes 6-13 days to incubate, but it can take up to 21 days (5). MPX is often self-limiting, although it can be severe in some people, including children, pregnant women, and individuals with immunosuppression due to other medical conditions. According to reports by the Centers for Disease Control and Prevention (CDC) and World Health Organization (WHO) 2022, homosexual men comprise a large number of cases. However, anyone in close contact with an infected individual is at high risk of infection.

**Figure 1:**
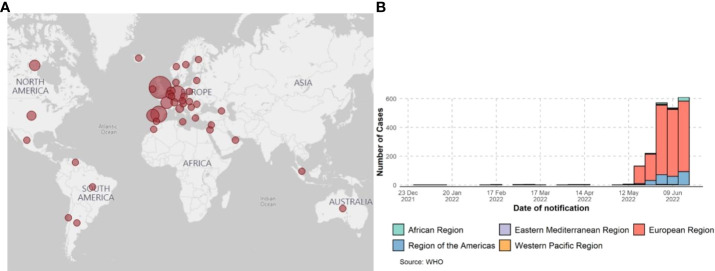
Global surveillance of MPX confirmed cases from January 2022 to 15 June 2022, data as of 15 June 2022. **(A)** CDC report; 2022 MPXV and Orthopoxvirus Outbreak Global Map - https://www.cdc.gov/. **(B)** Confirmed cases of MPXV by WHO report.

The first isolate of MPXV was identified in 1958 when monkeys shipped from Singapore fell sick in a research facility in Denmark (6). However, the first human case of MPX was confirmed in 1970 when the virus was isolated from a child suspected of having smallpox in the Democratic Republic of the Congo (7). Human MPX cases and sporadic clusters have also been reported outside of Africa. The first MPX outbreak in humans outside Africa was reported in 2003 in the midwest United States. This outbreak was linked to contact with prairie dogs housed in giant Gambian rats and dormice imported from Ghana. This has resulted in over 50 human cases of MPX in the U.S (8). A case of MPX was reported in a traveler from Nigeria to Israel in October 2018 (9). One case occurred in May 2019 when a man traveled from Nigeria to Singapore (10). Three members of a family traveling from Nigeria to the United Kingdom were reported to be infected with MPXV in May 2021 (11), one case was reported in July 2021 (Nigeria to Texas) (12), and one was reported in November 2021 (Nigeria to Maryland) (13). As of May 2022, clusters of human MPX have been reported in several non-endemic countries across the world (CDC and WHO, 2022) (**Figure 1**). This outbreak has been linked to a virus from the West African clade, which is commonly associated with milder symptoms, and in this case, human-to-human transmission has been suspected. Further investigations are underway to understand the epidemiology, sources of disease, and viral transmission patterns.

Vaccinia vaccination provided coincident immunity against MPXVs in the past; however, multiple observational studies have indicated approximately 85% efficiency in avoiding MPX. The eradication of smallpox following a lack of vaccine efforts has allowed MPX to gain clinical relevance. Currently, there are no clinically validated treatments for MPX infection (7). Therefore, new therapeutic strategies against emerging strains of the MPXV are urgently needed. The immune system plays a crucial role in pathogenesis as well as in the fight against viral infections and cancers. Advancements in immuno-informatics and bioinformatics techniques have facilitated the identification of novel therapeutic targets against a variety of pathogenic strains. Multi-epitope-based vaccination is an emerging strategy for the prevention of pathogenic diseases (14–17). The identification of immunogenic antigens is crucial for developing effective vaccines (18). Potent multi-epitope vaccine constructs have overlapping B- and T-cell epitopes in each antigenic peptide sequence, which induces cellular or humoral responses against target viral infections (14). In this study, we used reverse vaccinology and biophysical techniques to design a multi-epitope vaccine against MPXV infection. The entire protein sequence data of the latest 2022 strain of MPXV were used to identify lead B- and T-cell epitopes among the selected antigenic peptides. Overlapping lead epitopes have been used in the construction of chimeric vaccine structures. The efficacy of the designed vaccine constructs was evaluated using immuno-informatics, binding potential with immune receptor proteins, and *in silico* cloning into a host-vector expression system.

## Methodology

**Figure 2:**
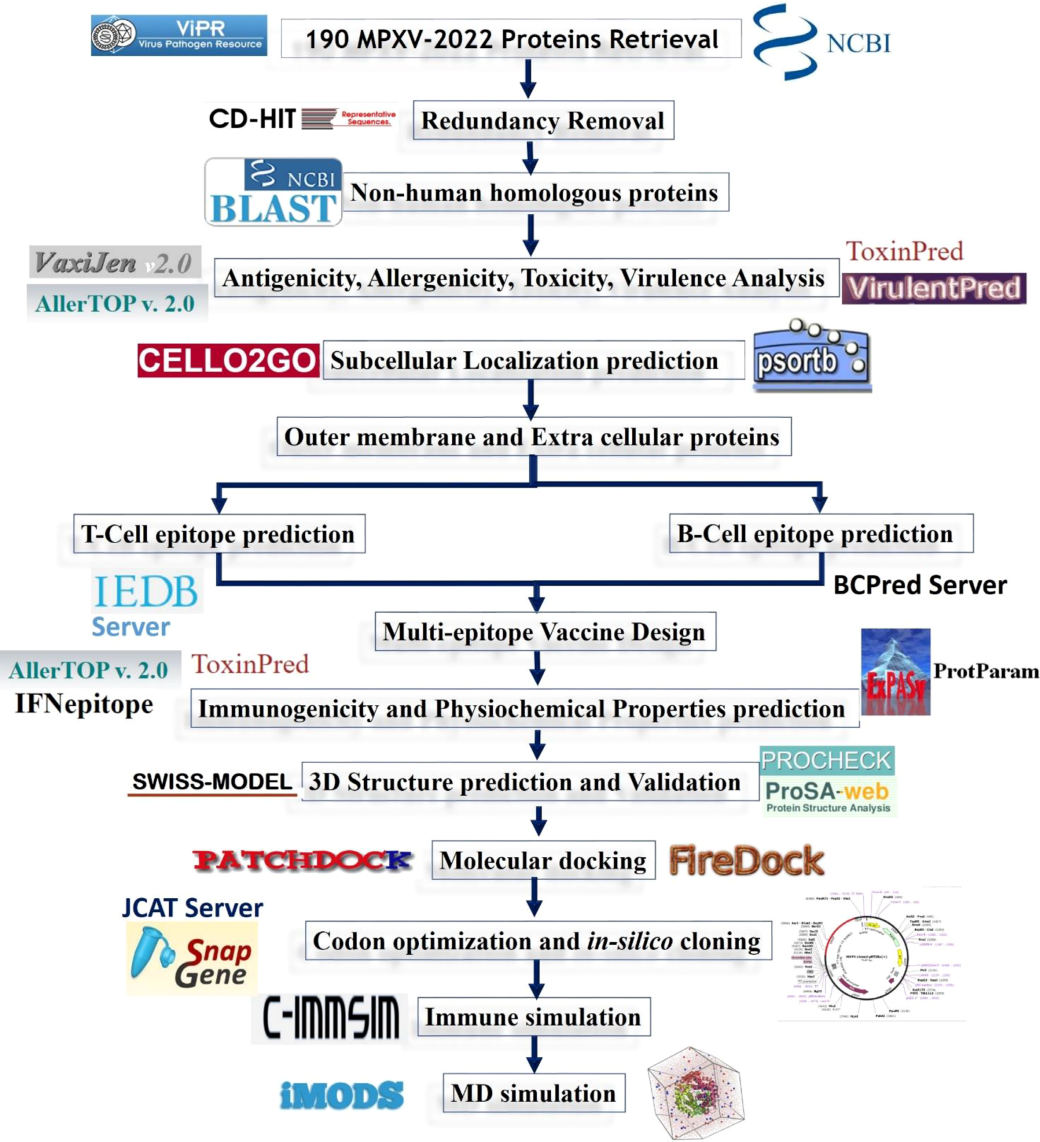
Systematic workflow of the designed study.

The systematic workflow followed in the current study to design a multi-epitope vaccine construct against monkeypox is shown in **Figure 2**.

### Proteins sequence retrieval

All proteins of the MPXV_USA_2022_FL001 strain were retrieved from the virus pathogen resource (VIPR) database (19) and confirmed with NCBI data entry. The entire amino acid sequences of the proteins were obtained in FASTA format based on data submitted to the NCBI in May 2022 with humans as the host of the virus. The CD-hit suite was used to remove redundancy and obtain non-paralogous protein sequences with a threshold of 80% sequence similarity (20). Sequences homologous to human proteins were removed using the NCBI-BLASTp program (21) with a threshold of setting an e-value cut-off of 10^−4^, percent identity ≤ 35, query coverage ≤ 70, bit score ≤ 100, and the rest of the parameters were set as default. AllerTOP2.0 web tool was used to determine the allergenicity of non-human homologous viral proteins (22). The antigenicity of the selected proteins was evaluated using the VaxiJen v 2.0 web server with a threshold of above 0.4 (23). The ToxinPred web server was used to assess the toxicity of shortlisted viral proteins (24). The virulence potentials of antigenic, non-toxic, and non-allergenic proteins were examined using the Virulentpred web tool (25). Non-human homologous viral proteins were subjected to subcellular localization using PSORTb version 3.0.2 (26) and CELLO2GO V.2.5 (27) web servers. Outer-membrane and extracellular proteins were selected as suitable vaccine candidates (17).

### T-Cell and B- Cell epitopes prediction

Proteins located in the outer membrane and extracellular region were selected as suitable vaccine candidates. The surface topology of these proteins was chosen to identify the immunogenic determinants (epitopes) for chimeric vaccine design. T cell epitopes are represented by major histocompatibility complex (MHC) molecules as class I (MHC-I) and II (MHC-II), which are recognized by two separate subsets of T-cells, CD8, and CD4, respectively (28). T-cell epitopes of the selected protein sequences were predicted using the Immune Epitope Database (IEDB) (29). The stabilized matrix method (SMM) scoring method and a neutral network-based tool (net MHC-1.1) were utilized for MHC-I and MHC-II prediction. The top binding overlapping epitopes with a calling criterion of IC_50_ 200 nM and a length of 12-20 residues were prioritized (21, 30). The BCpred server was used to predict B-cell epitopes with the cut-off value set at > 0.8 and other default parameters. BCpred identifies linear B-cell epitopes that are critical for inducing a humoral immune response that stimulates B lymphocytes to produce antibodies (31). The IFNepitope web server was used to identify interferon-inducing epitopes from MHC-II binding epitopes. The IFNepitope server predicts the region of antigenic protein sequences that causes interferon-gamma (IFN-γ) induction. IFN-γ was predicted using the SVM model, and IFN-gamma vs. non-IFN-gamma model was selected for prediction (32). Epitopes positive for IFN-γ inducers were selected for further analysis.

### Epitope selection and vaccine design

Peptide vaccines are typically weak immunogens; however, integrating immunodominant epitopes to form a multi-epitope peptide vaccine can improve immunogenicity. B- and T-cell epitopes are immunodominant and crucial for inducing strong immunogenic responses to toxins and contagions (33). Overlapping B-cell, MHC-I, MHC-II, and IFN epitopes were selected based on cut-off values and manual comparisons. The rationale behind selecting overlapping B- and T-cell epitopes was to ensure that the designed vaccine could generate both humoral and cytotoxic immune responses (30). Effective synthesis of vaccine constructs is affected by variations in the expression and distribution of HLA alleles across different regions and ethnicities worldwide (34). The IEDB population coverage tool (http://tools.iedb.org/population/) was used to calculate the population coverage of the selected MHC-I and MHC-II epitopes and the associated HLA-binding alleles. Based on the distribution of HLA binding alleles, this tool calculates the population coverage of each epitope in various regions around the world (35). The IEDB epitope conservancy tool (http://tools.iedb.org/conservancy/) was used to determine the conservation of selected epitopes among various strains of MPXV.

A multi-epitope vaccine construct is also comprised of a strong immunostimulatory adjuvant to improve immunogenicity and activate long-term innate and adaptive immune responses (36). Four different adjuvants (HBHA protein, β-defensin, 50S ribosomal protein L7/L12 adjuvants, and HBHA conserved peptide sequences) were incorporated into the design of the multi-epitope vaccine. EAAAK linkers were used to join adjuvant sequences at the N-terminus of the vaccine constructs. HEYGAEALERAG and GGGS linkers were used to join the final epitopes (17). The EAAAK linker was used as a stiff spacer to bind the N terminus of the adjuvant to the epitope. HEYGAEALERAG and GGGS linkers are based on the method of Solanki & Tiwari (2018) as flexible linkers. All of these linkers are used for the best expression, bioactivity improvement, and to generate high immunogenic responses by the designed vaccine (37).

### Immunological and physiochemical properties

Various physicochemical properties of the vaccine constructs were evaluated using the ProtParam tool on the ExPASy server (38). The multi-epitope vaccine constructs were subjected to immunogenic analysis. The allergenicity of the vaccine constructs was predicted using the AllerTOP2.0 web tool (22). The antigenicity of the vaccine constructs was determined using the VaxiJen v2.0 web server with a threshold of above 0.4 (23). The SOLpro web server was used to predict the solubility of vaccine constructs (39). The toxicity of the vaccine constructs was evaluated using the ToxinPred server (40).

### Homology modeling, 3D structure validation, and molecular docking

The three-dimensional structures of the multi-epitope vaccine constructs were predicted using the Swiss Model server (41). The predicted vaccine structures were refined using the DeepRefiner web server (42). The refined 3D structures of vaccine constructs were further validated using the ERRAT tool, PROCHECK suite of programs (43) of structure validation server SAVES v6.0 (https://saves.mbi.ucla.edu/) and ProSA-Web (https://prosa.services.came.sbg.ac.at/prosa.php). Understanding the binding pattern between designed vaccines and the TLR4 immune cell receptor is crucial to generate successful immune responses (44). The vaccine constructs were docked into the human TLR4 receptor (PDB ID: 4G8A) (45) using the PatchDock web server to evaluate immune receptor-vaccine interactions (46). All heteroatoms and cocrystallized ligands were removed from the TLR4 3D structure using Molecular Operating Environment (MOE) software for receptor preparation and preprocessing (47). The Fire Dock (Fast Interaction Refinement in Molecular Docking) server was used to refine the results of the molecular docking (48).

### Codon optimization and *in silico* cloning

Codon optimization and *in silico* cloning were performed for the final vaccine construct (V2). The Java Codon Adaptation (JCat) tool was used for reverse translation and codon optimization of the vaccine construct to obtain higher expression of the cloned sequence in the *E. coli* expression system (49). JCat provides the percentage of GC content and codon adaptation index (CAI) to evaluate the expression potential of the cloned vaccine sequence. For favorable transcriptional and translational efficacy, the optimum value reported for CAI is 1 and the GC content is 30%-70% (17, 45). Finally, the Snapgene tool (https://www.snapgene.com/) was used for restriction cloning of the optimized vaccine construct in the *E. coli* expression system. pET28a_TIAL1 (*E. coli* plasmid) was retrieved from the Addgene server (https://www.addgene.org/).

### Immune simulation

Computational immune simulation of the finalized multi-epitope vaccine construct was performed using the C-ImmSim server to evaluate the immunogenic potential of the vaccine (50). The server uses a position-specific score matrix (PSSM) along with various machine learning approaches to predict multi-epitope vaccines and immunological interactions. It is a cellular-level agent-based model that obtains information about humoral and cellular responses of the mammalian immune system in response to antigens (51). Immune simulation performed for the vaccine construct was based on a protocol previously described by Bibi et al.  (15). The simulation parameters were set as default for a period of 1 h, 84 h, and 168 h along with human host leukocyte antigens selection (HLA-A*0101 & A*0201, HLA-B*0702 & B*3901, and HLA-DRB1*0101 & DRB1*0401). The volume of the simulation was set at 10 and random seed at 12345 with vaccine injection not containing LPS. An immune simulation was conducted for 1000 simulation steps.

### Molecular dynamic simulation

A molecular dynamics (MD) simulation study was conducted for the MPXV-V2-TLR4 complex with the best docking analysis results. The iMODS web server was used for MD simulations, energy minimization, and calculation of protein flexibility (52). iMODS is based on normal mode analysis (NMA) in the internal (dihedral) coordinates of macromolecules that naturally reproduce the collective functional movements of biological macromolecules. iMODS generates practical transition pathways between two homologous structures of the macromolecules based on these modes. The server defines potential conformational changes, detects elastic network potentials, models resolution with numerous coarse-grained atomic representations, and provides an improved affine-model-based arrow representation of the complex domain dynamics of macromolecules. The server investigates the structural dynamics of proteins and docked protein complexes with other proteins and ligands to provide values of deformability, eigenvalues, variance, B-factor (mobility profiles), covariance map, and elastic network data based on NMA (53). The docked PDB file for the MPXV-V2-TLR4 complex was submitted to the iMODS server and the results were obtained based on the default settings for all parameters.

## Results

### Proteins sequence retrieval

**Figure 3:**
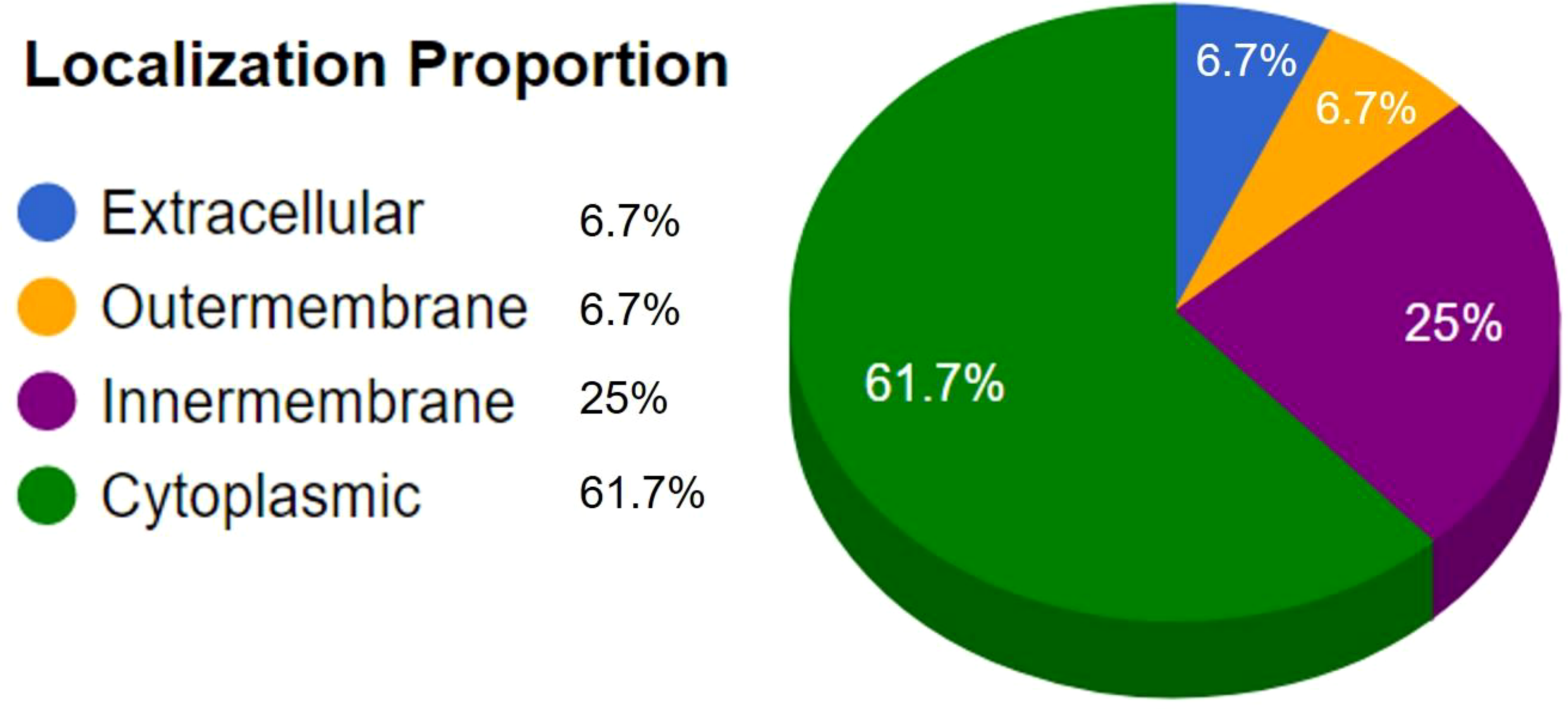
Subcellular localization results predicted by CELLO2GO.

All the proteins available for the latest strain of MPXV retrieved from the VIPR database yielded 190 protein sequences with NCBI and GenBank information (**Supplementary file 1**). Duplicate sequences were removed, and 186 non-paralogous sequences were obtained using CD-Hit recourse with a threshold of 80% sequence similarity (**Supplementary file 2**). A total of 43 sequences remained after the removal of human-homologous sequences. These proteins were subjected to further analysis based on antigenicity, allergenicity, toxicity, and virulence (**Table S1**). The subcellular localization identified three proteins, URP85109, URP84966, and URP85049, in the outer membrane and extracellular region to be prioritized as vaccine candidates (**Figure 3**; **Table 1**). Based on VaxiJen (23) prediction, the finalized proteins were highly antigenic with prediction scores of 0.5734, 0.5992, and 0.5316 respectively, at a threshold of >0.4. Moreover, the prioritized vaccine candidate proteins are non-allergenic, non-toxic, and involved in virulence, implying that host cell-induced immunogenic responses target only the virus rather than the host (40).

**Table 1:** Subcellular localization results predicted by PSORTb web server.

Position	Number of proteins
Cytoplasmic	27
Unknown	16

### T-cell and B-cell epitopes prediction

**Figure 4:**
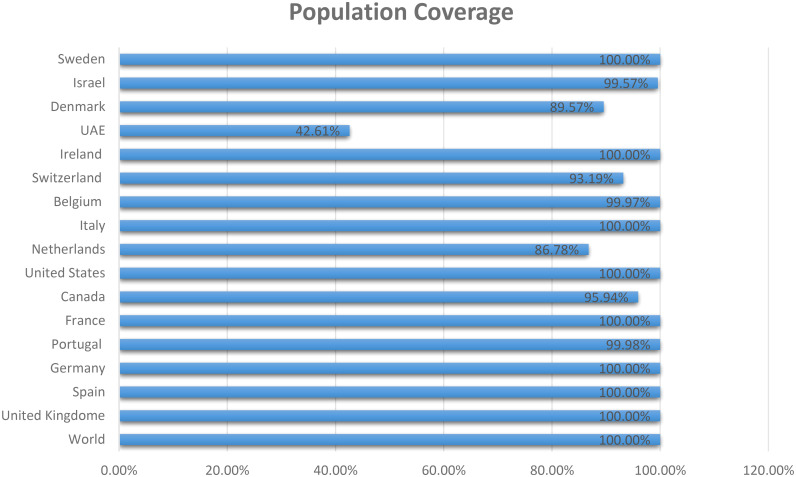
Population coverage of MHC epitopes in highly infected regions of the world calculated by IEDB.

The three proteins prioritized in this study were subjected to further analysis to identify lead epitopes for designing chimeric vaccine constructs against MPXV. T-cell (MHC-I and MHC-II) epitopes for the selected proteins were predicted based on an IC_50_ threshold of < 200 nM using the IEDB server. Overlapping B-cell epitopes were predicted with BCpred scores > 0.8 and 75% specificity. Three overlapping lead epitopes were predicted for each prioritized protein which was used to design vaccine constructs. Nine epitopes were prioritized based on their high antigenicity, IFN-positivity, low toxicity, and low allergenic reactions (**Table 2**). The ultimate objective was to identify lead epitopes with the capability to induce humoral and cell-mediated immunogenic responses and host interferons. The conservation of the selected epitopes was confirmed in various strains of MPXV. The use of conserved epitopes in the multi-epitope vaccine construct would provide broader protection against different strains of MPXV (54). The conservation of the selected epitopes is presented in **Table 3**. The selected epitopes demonstrated a 100% coverage of the global population (**Table 4**). The IEDB results revealed that the population coverage of the predicted epitopes was high in countries most affected by MPXV, including European countries, Israel, Canada, and the United States (**Figure 4**).

**Table 2:** T-cell and B-cell epitope prediction showing overlapping epitopes, IFN-γ epitope identification, allergenicity, and toxicity analysis.

Protein IDs	MHC-I Epitopes	MHC-II Epitopes	IC_50_ ≤200	B-Cell Epitopes	BCPred Score	IFN-GamaPositive Score	Allergenicity-AllerTOP2.0	Toxicity
**URP85109**	RSNEEFDPV	GKWNPILPTCVRSNE	83	SNEEFDPVDDGPVSDYVSELY	0.975	0.55616266	Non allergen	Non-Toxin
	AVVYSTCTV	TLLCVLPAVVYSTCT	38	LPAVVYSTCTVPTMNNAKLT	0.975	1.343031	Non allergen	Non-Toxin
	YISCTANSW	VIGVSYISCTANSWN	191	YISCTANSWNVIPSCQQKCD	0.842	1.3838816	Non allergen	Non-Toxin
**URP84966**	KINSIVERR	LNFRQDAVNKINSIV	23	NKINSIVERRSGMSNVVDST	0.942	0.53707317	Non allergen	Non-Toxin
	TVAEASTIM	VAEASTIMVATARSS	143	VAEASTIMVATARSSPEELE	0.904	0.40167514	Non allergen	Non-Toxin
	PMMNVVTKL	MMNVVTKLQGNTITI	62	TKTVPMMNVVTKLQGNTITI	0.831	0.45337431	Non allergen	Non-Toxin
**URP85049**	LVHWNKKKY	GEINLVHWNKKKYSS	78	VHWNKKKYSSYEEAKKHDDG	0.966	0.59602987	Non allergen	Non-Toxin
	SNHEGKPHY	KFRTLLSSSNHEGKP	86	SSSNHEGKPHYITENYRNPY	0.891	0.62107689	Non allergen	Non-Toxin
	GFLPNEYVL	GGFLPNEYVLSTIHI	117	VRINFKGGYISGGFLPNEYV	0.851	0.63304856	Non allergen	Non-Toxin

Red indicates overlapping amino acid residues in the MHC-I, MHC-II and B-cell epitopes.

**Table 3:** Epitope conservancy of the selected B- and T- cell epitopes calculated by IEDB.

Protein IDs	MHC-I Epitopes	Conservation	MHC-II Epitopes	Conservation	B-Cell Epitopes	Conservation
**URP85109**	RSNEEFDPV	0.47% (1/213)	GKWNPILPTCVRSNE	0.47% (1/213)	SNEEFDPVDDGPVSDYVSELY	0.00% (0/213)
	AVVYSTCTV	0.47% (1/213)	TLLCVLPAVVYSTCT	0.47% (1/213)	LPAVVYSTCTVPTMNNAKLT	0.47% (1/213)
	YISCTANSW	0.47% (1/213)	VIGVSYISCTANSWN	0.47% (1/213)	YISCTANSWNVIPSCQQKCD	0.47% (1/213)
**URP84966**	KINSIVERR	0.47% (1/213)	LNFRQDAVNKINSIV	0.47% (1/213)	NKINSIVERRSGMSNVVDST	0.47% (1/213)
	TVAEASTIM	0.47% (1/213)	VAEASTIMVATARSS	0.47% (1/213)	VAEASTIMVATARSSPEELE	0.47% (1/213)
	PMMNVVTKL	0.47% (1/213)	MMNVVTKLQGNTITI	0.47% (1/213)	TKTVPMMNVVTKLQGNTITI	0.47% (1/213)
**URP85049**	LVHWNKKKY	0.47% (1/213)	GEINLVHWNKKKYSS	0.47% (1/213)	VHWNKKKYSSYEEAKKHDDG	0.47% (1/213)
	SNHEGKPHY	0.47% (1/213)	KFRTLLSSSNHEGKP	0.47% (1/213)	SSSNHEGKPHYITENYRNPY	0.47% (1/213)
	GFLPNEYVL	0.47% (1/213)	GGFLPNEYVLSTIHI	0.47% (1/213)	VRINFKGGYISGGFLPNEYV	0.47% (1/213)

**Table 4:** Population coverage of MHC epitopes in different regions across the world calculated by IEDB.

Region	URP85109		URP84966		URP85049	
	MHC-I Epitopes	MHC-II Epitopes	MHC-I Epitopes	MHC-II Epitopes	MHC-I Epitopes	MHC-II Epitopes
World	100.0%	83.81%	100.0%	83.81%	100.0%	83.81%
East Asia	100.0%	82.41%	100.0%	82.41%	100.0%	82.41%
Northwest Asia	98.55%	60.8%	98.55%	60.8%	98.55%	60.8%
South Asia	100.0%	76.44%	100.0%	76.44%	100.0%	76.44%
Southeast Asia	100.0%	58.83%	100.0%	58.83%	100.0%	58.83%
Southwest Asia	98.66%	45.29%	98.66%	45.29%	98.66%	45.29%
Europe	100.0%	87.47%	100.0%	87.47%	100.0%	87.47%
East Africa	98.74%	68.53%	98.74%	68.53%	98.74%	68.53%
West Africa	99.12%	65.81%	99.12%	65.81%	99.12%	65.81%
Central Africa	98.39%	62.84%	98.39%	62.84%	98.39%	62.84%
North Africa	99.6%	76.07%	99.6%	76.07%	99.6%	76.07%
South Africa	99.64%	32.1%	99.64%	32.1%	99.64%	32.1%
West Indies	99.57%	70.02%	99.57%	70.02%	99.57%	70.02%
North America	100.0%	90.03%	100.0%	90.03%	100.0%	90.03%
Central America	9.07%	53.91%	9.07%	53.91%	9.07%	53.91%
South America	100.0%	63.52%	100.0%	63.52%	100.0%	63.52%
Oceania	99.15%	60.21%	99.15%	60.21%	99.15%	60.21%

### Multi-epitope chimeric vaccine constructs

Several combinations of selected lead epitopes were used to design a chimeric vaccine construct. The epitopes were linked using GGGS and HEYGAEALERAG linkers. Linkers provide stability to vaccine constructs and functional prevention of each epitope, allowing them to function independently after injection into the human host (55). The epitopes were linked to four different adjuvants: HBHA protein, beta-defensin, 50S ribosomal protein L7/L12 adjuvants, and HBHA conserved peptide sequences at the N-terminal with the help of EAAAK linkers to boost immunogenic responses. Moreover, PADRE peptide sequences have been incorporated into the designed vaccine constructs to avoid complications caused by HLA-DR variation in different populations worldwide. Previous studies have reported that vaccine constructs incorporating the PADRE peptide exhibit enhanced immune protection and high cytotoxic T lymphocyte (CTL) responses (56). The immunological properties of the vaccine constructs showed that all four constructs were non-allergenic and non-toxic. Antigenicity scores > 0.9, calculated by the ANTIGENpro server indicate the substantial antigenic nature of the multi-epitope vaccine constructs. ANTIGENpro evaluates antigenicity by 10-fold cross-validation of peptide sequences based on known datasets and identification of protective aspects of the antigenic sequences (57). VaxiJen 2.0 scores for all constructs ranged from 0.44 to 0.47, which is equal to the default threshold typically used for viruses. SOLpred scores > 0.97 indicated high solubility of the vaccine constructs upon expression (**Table S2**). The physiochemical properties of the vaccine constructs calculated by the ProtParam server indicated that the molecular weights of all these constructs ranged from ~41 kDa to ~54 kDa. GRAVY scores from -0.189 to -0.378 indicate the hydrophilic nature of the designed vaccine constructs. The theoretical pI values were in the range of 5.25 to 8.74. Aliphatic index scores ranged from 64.81 to 75.10, which indicates the thermostability of these constructs. The instability index scores were predicted to range from 30 to 40, indicating the stability of these constructs at various temperatures (**Table 5**). No significant changes were observed in the physicochemical properties of the constructs, as the amino acid content for all these constructs was similar, with the only difference being the adjuvant. Immunogenic and physiochemical property analysis signified the capability of vaccine constructs to initiate a significant immunogenic response within the human host. However, further experimental research is required to verify the accuracy of these results.

**Table 5:** Physiochemical properties of the vaccine constructs using ProtParam server and JCAT server.

Vaccine construct	Number of Amino Acids	Molecular Weight (Daltons)	Theoretical pI	Aliphatic index	Grand average of hydropathicity (GRAVY)	Instability index	GC content	CAI
**MPXV-V1**	511	54123.10	5.35	72.78	-0.378	36.86 (stable)	71.88	0.96
**MPXV-V2**	397	41655.65	8.74	64.81	-0.368	34.92 (stable)	70.69	0.95
**MPXV-V3**	502	53004.87	5.25	74.64	-0.356	40.00 (stable)	71.64	0.95
**MPXV-V4**	482	49934.94	5.37	75.10	-0.189	30.03 (stable)	70.88	0.96

### 3D structure prediction, validation, and molecular docking

**Figure 5:**
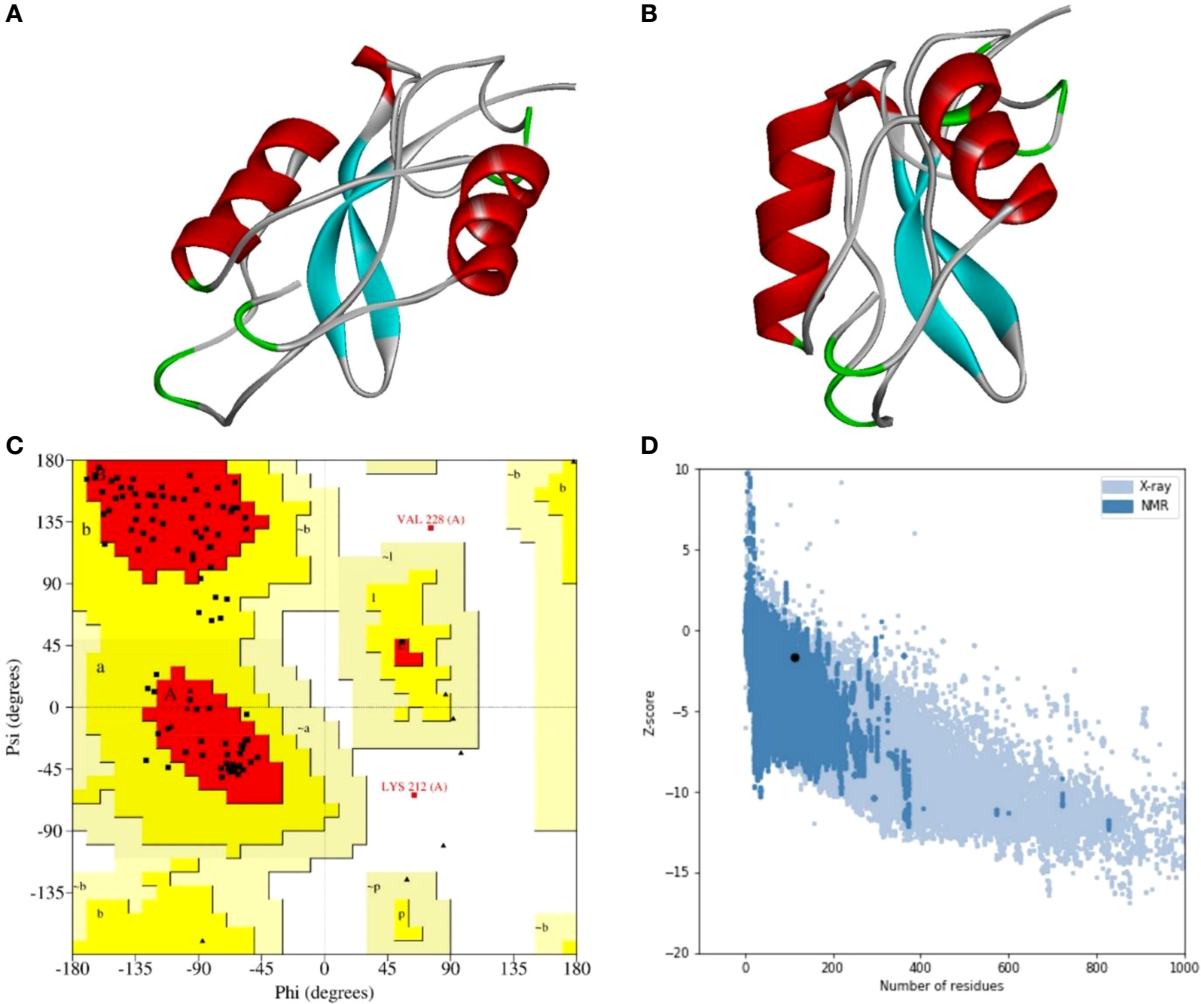
Three-dimensional structure analysis, structure refinement, and structure validation of MPXV-V2. **(A)** The 3D model of the multi-epitope vaccine was built by the Swiss Model using a homology modeling approach. **(B)** Refine 3D structure of MPXV-V2 obtained from DeepRefiner web-server. **(C)** Ramachandran plot analysis shows 84.5% residues in the favored region, 13.4% residues in the allowed region, and 2.1% residues in the disallowed region of the plot. **(D)** ProSA-web results with a Z-score of -1.66.

**Figure 6:**
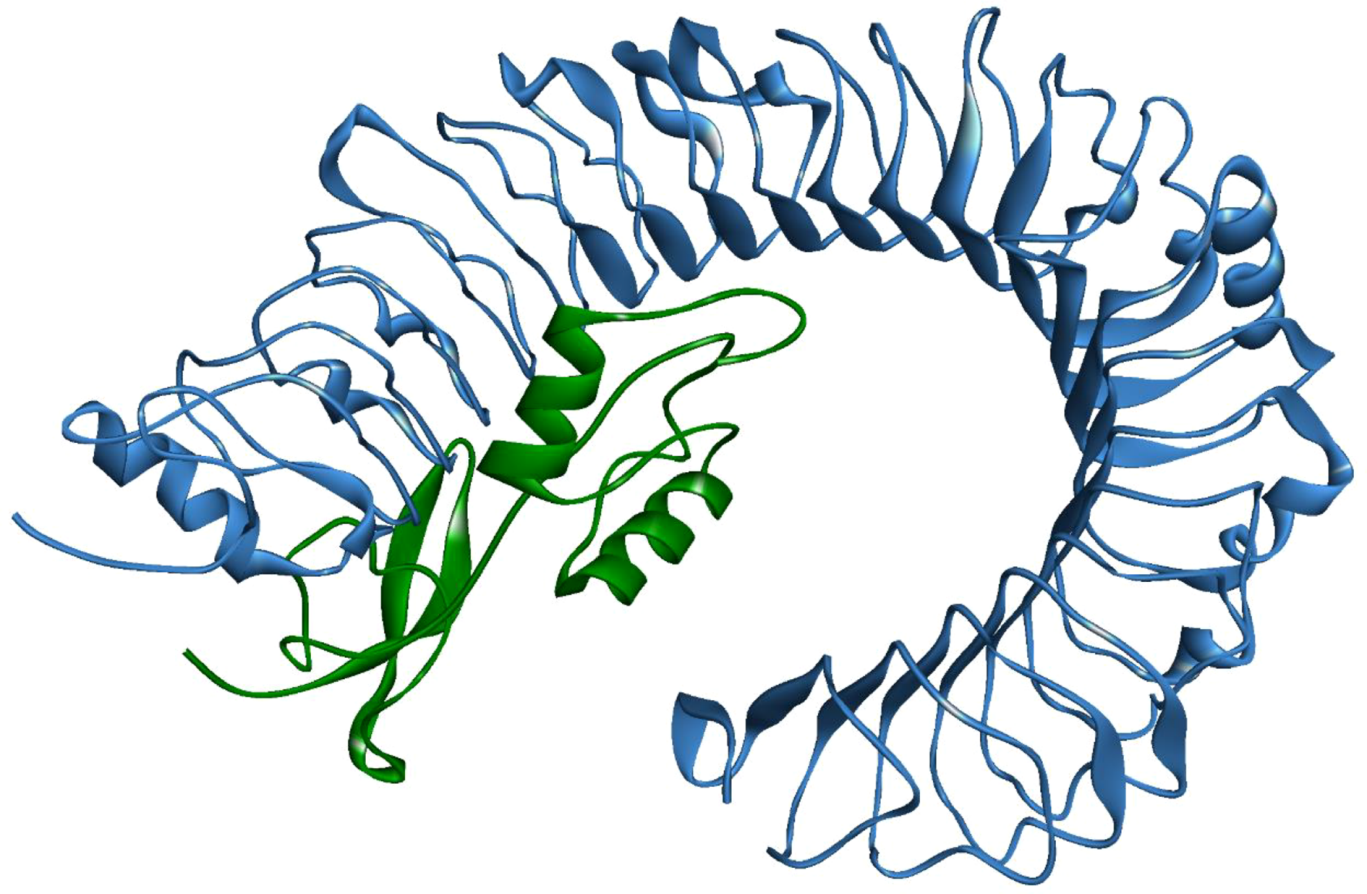
MPXV-V2 docked complex with TLR4 receptor. Blue indicates TLR4 and Green indicates the vaccine construct.

A stable and functional three-dimensional structure of a vaccine is crucial for studying its molecular interactions with immune receptor proteins (58). Prediction of the vaccine constructs was anticipated by homology modeling approaches using the Swiss Model server (**Figure 5A**), refined by the DeepRefiner web server, and subjected to structural validation analysis (**Figure 5B**). The Ramachandran plots of the vaccine constructs revealed that 99.0% residues of V1 (HBHA), 84.5% residues of V2 (beta-defensin construct), 84.5% residues of V3 (HBHA conserved), and 89.4% residues of V4 (50S ribosomal) appeared in the favored region of the plots (**Figure 5C**). With the ERRAT, the overall quality factor of the refined vaccine constructs ranged from 85% to 100%. Similarly, the ProSA-web server evaluated the Z score for the vaccine constructs to be -5.94 to -1.66 (**Figure 5D**). The overall results from RAMPAGE, the ERRAT web tool, and the ProSa-web server determined the excellent quality of the designed vaccine 3D structures (**Table 6**). Molecular docking is an efficient method for identifying the optimal binding between designed vaccine constructs and receptor molecules. The PatchDock server (a blind docking technique) was used to dock the vaccine constructs with the surface human TLR4 immune receptor. The top ten results were further subjected to the FireDock web server to refine the docked complexes. FireDock resolves the protein flexibility issues that may occur during protein-peptide docking, enabling high-throughput complex refinement. The docking scores of all the vaccine-receptor complexes were similar. However, the V2 construct (i.e. Beta defensin adjuvant) showed the lowest binding energy (-12.08 kcal/mol) with the TLR4 receptor during this study (**Figure 6**). The docking analysis revealed strong binding affinities between the vaccine constructs and the TLR4 receptor (**Table 7**).

**Table 6:** 3D structural validation of vaccine constructs *via* ERRAT, PROCHECK (Ramachandran plot favoured region), and ProSA-Web Server.

Vaccine construct	Constructs	ERRAT	PROCHECK	ProSA (Z- score)
**MPXV-V1**	V1	100	99.0%	-3.43
**MPXV-V2**	V2	93.3333	84.5%	-1.66
**MPXV-V3**	V3	85.5072	84.5%	-1.85
**MPXV-V4**	V4	97.479	89.4%	-5.94

**Table 7:** Docking score, the interface area, the contribution of the hydrogen bonds, global energy, and atomic contact residues energy of vaccine constructs in HLA and TLR4 molecules.

Vaccine construct	Score	Area	HB-Contribution	Global energy	Atomic Contact residues energy (ACE)
**MPXV-V1-TLR4**	14006	1958.70	-3.22	-1.46	23.99
**MPXV-V2-TLR4**	14690	1937.40	-0.93	-12.08	1.95
**MPXV-V3-TLR4**	14844	1967.00	-6.02	-10.78	18.30
**MPXV-V4-TLR4**	13984	1891.90	-0.29	0.97	3.31

### Codon optimization and *in silico* cloning

**Figure 7:**
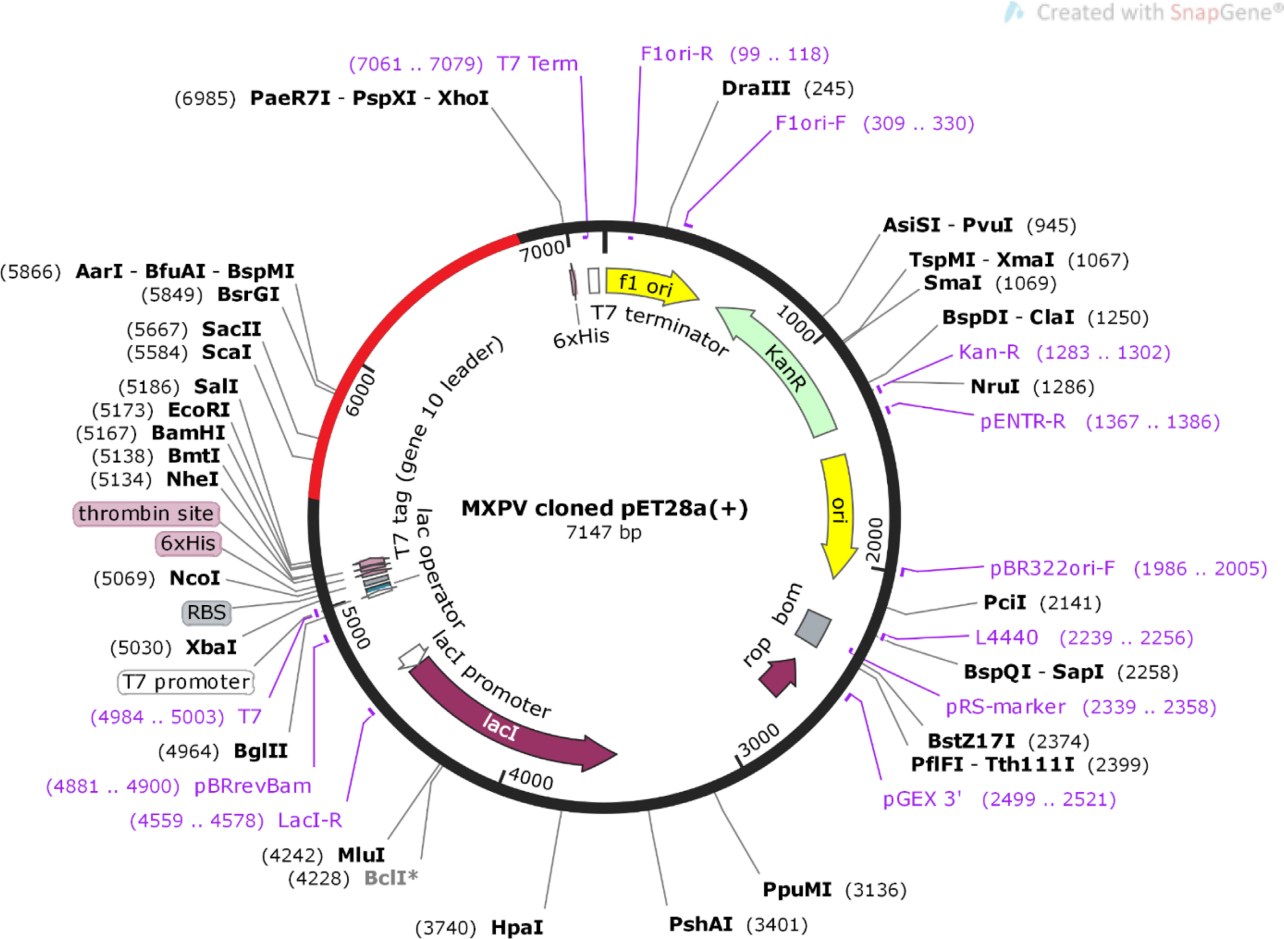
*In silico* restricted cloning of MPXV-V2 vaccine into *E.coli* expression vector pET28a (+).

The sequences of the designed vaccine constructs were subjected to codon optimization using the JCat web server. The peptide sequences were reverse-translated into DNA sequences to achieve higher expression levels of the vaccine constructs in the *E. coli* expression system. The CAI values for all constructs were predicted to be~0.95, and the average GC content of the adapted sequences was ~70% indicating an acceptable range for higher expression of the designed vaccine in the *E. coli* host (**Tables 4**; **Table S3**). Finally, SnapGene software was used to construct a recombinant plasmid sequence by introducing the adapted codon sequence of the final vaccine construct V2 into the plasmid vector pET28a(+), thereby ensuring heterologous cloning and expression in the *E. coli* system (**Figure 7**).

### Immune simulation

**Figure 8:**
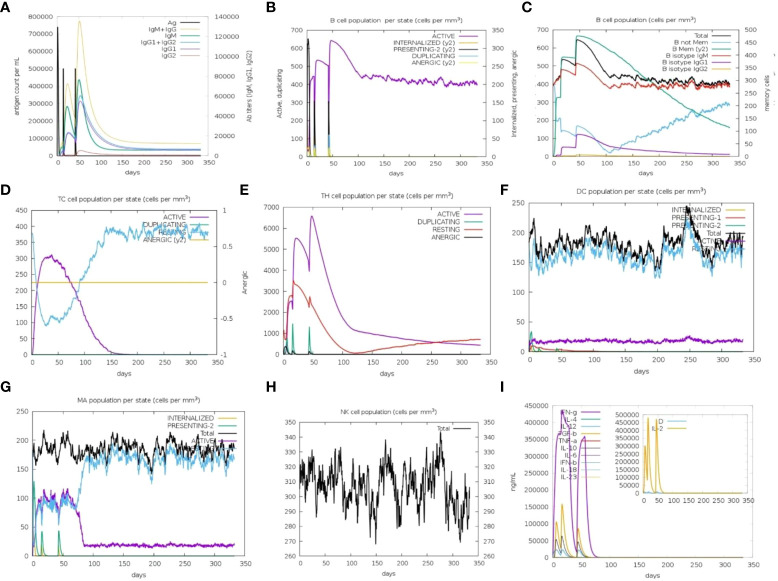
*In silico* immune simulation to predict the immunological potential of designed vaccine MPXV-V2 chimeric peptide predicted by C-ImmSim Server. **(A)** Increased level of immunoglobin antibodies with a decrease in antigen levels upon vaccine injections. **(B)** Increased levels of B-cell populations with a decrease in antigen levels upon vaccine injections. **(C)** Rising B-cell populations after repeated exposure to antigen. **(D, E)** The increase in the population of T-cytotoxic and T-helper cells upon repeated antigen exposure. **(F–H)** The population increase of dendritic cells, macrophages, and natural killer cells during the immunization period. **(I)** Increased concentrations of cytokine and interleukin levels after the repeated antigen exposure. The inset plot shows the danger signal together with leukocyte growth factor IL-2.

Immune simulation predictions resulted in a significant increase in the secondary responses induced by the prioritized vaccine construct. In principle, this trend is consistent with the development of real-time immune responses. Elevated levels of IgM were the primary simulated response. The secondary and tertiary simulated responses exhibited significant increases in B-cell populations, as well as high levels of IgG1 + IgG2, IgM, and IgM + IgG antibodies. However, a decrease in the antigen levels was observed (**Figures 8A, B**). This suggests the generation of immunological memory, as evidenced by the increased level of memory B-cell population along with isotype switching. This led to a rapid antigen decrease following the subsequent chimeric antigen exposure (**Figure 8C**). The cytotoxic (TC) and helper (TH) T-cell populations were predicted to have a similar higher response, with the development of corresponding memory upon subsequent antigen exposure (**Figures 8D, E**). Additionally, during the immunization period, macrophage, dendritic cell, and natural killer cell populations were triggered and maintained at elevated levels (**Figures 8F–H**). Increased concentrations of cytokines, such as IFN-y, and interleukins, such as IL-2, were also observed (**Figure 8I**). These findings suggest that the predicted vaccine construct elicited encouraging immune responses against MPXV.

### Molecular dynamic simulation

**Figure 9:**
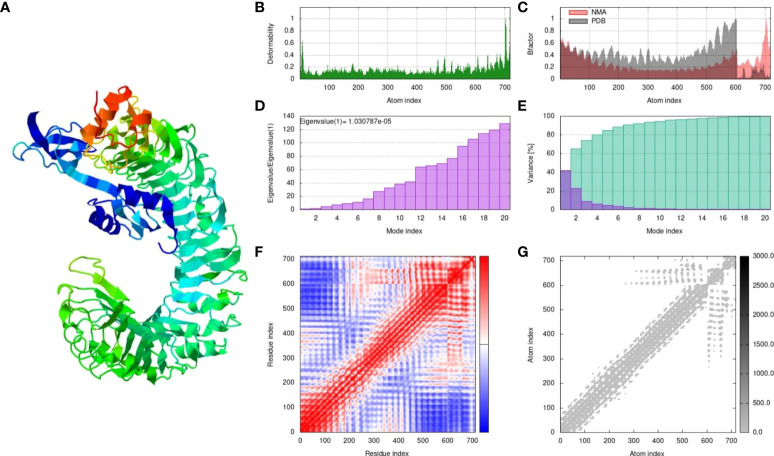
The results of molecular dynamics simulation of vaccine MPXV-V2 and TLR4 complex achieved by iMODS server. **(A)** NMA mobility, **(B)** deformability, **(C)** B-factor which indicates an averaged RMS, **(D)** eigenvalues **(E)** colored bars show the individual (purple) and cumulative (green) variances **(F)** Covariance matrix indicates correlated (red), uncorrelated (white), and anti-correlated (blue) motions of paired residues and **(G)** The elastic network model (grey regions indicates stiffer regions).

The MPXV-V2 construct was finalized based on the lowest global energy upon docking with the TLR4 receptor. MPXV-V2-TLR4 complex was subjected to MD simulation for energy minimization and protein stability analysis. A simulation analysis was performed to determine the movement of atoms and molecules in the designed vaccine within a biological system using the iMODS webserver. iMODS uses a normal mode analysis approach to describe the collective functional motion of macromolecules. Each normal mode comprises a frequency that is correlated with the relative motion amplitude, and a deformation vector that specifies the direction of an atomic displacement of the macromolecules to determine the molecular flexibility of these molecules within the cellular environment (59). The results of the MD simulation and normal mode analysis (NMA) of the vaccine MPXV-V2 and TLR4 docked complex are shown in **Figure 9A**. To simulate possible transitions, the input structure was deformed iteratively along the lowest modes, and the RMSD of the target structure was minimized based on the local and global superposition of the structures. Main-chain deformability is a measure of the atomic displacements summed over all modes of residues at every atomic position. The deformability graph of the complex depicts the peaks that indicate deformable regions of the protein. The flexible regions (hinges/linkers) of the chain have high values, and the lower values are usually in the rigid regions of the main chain residues. The deformability graph of the complex shows the peaks in the graphs that correlate to the deformed regions of the protein (**Figure 9B**). The NMA-derived B-factor determines the relative amplitude of atomic displacements of the molecular complex around equilibrium confirmation. The B-factor graph illustrates the relationship between the mobility of the docked complex NMA and the PDB scores (represents the average RMSD) (**Figure 9C**). The eigenvalue associated with each normal mode represents the motion stiffness. This value is directly related to the energy required to deform a structure. The lower the eigenvalue, the easier the deformation of the carbon alpha atoms. The eigenvalue of the MPXV-V2 and TLR4 complex was 1.030787e-05, which represented the stability of the complex (**Figure 9D**). The variance graph is associated with each normal mode of the complex representing individual (purple) and cumulative (green) variances and is inversely related to the eigenvalue (**Figure 9E**). The covariance map of the complex indicates coupling between pairs of residues in the system. Covariance analysis is used to describe the correlated (red), uncorrelated (white), or anti-correlated (blue) atomic movements in the dynamical regions of the complex molecule (**Figure 9F**). The correlation matrix was computed using the Cα Cartesian coordinates and Equation 2 by Ichiye and Karplus 1991 (60). The elastic network model of the complex defines the relationship between the atoms. Each dot in the graph represents a spring that connects the corresponding pair of atoms. Dots are colored according to their stiffness, and the darker greys imply stiffer regions, whereas the lighter dots indicate flexible regions (**Figure 9G**).

## Discussion

Prevention of epidemic MPXV is challenging because of the reported increase in cases of human MPX and sporadic clusters worldwide. The available vaccines offer limited protection against MPX, especially in children and adults with underlying health conditions (7). Therefore, novel therapeutic strategies are required for emerging MPXV infections. Advancements in reverse vaccinology, as well as the availability of genomic and proteomic data, have assisted in vaccine design. Furthermore, the implementation of advanced bioinformatics tools is more beneficial than traditional approaches (61). Epitope-based vaccines are a novel therapeutic approach for the design and development of suitable vaccines with high potency, logistic viability, and improved safety. Multi-epitope vaccines have the potential to generate specific immunogenic responses based on conserved epitopes in complete antigenic sequences, thus avoiding responses against unfavorable epitopes that might induce immunopathogenic or immune-modulating responses against the host (62, 63). To date, there is no specific treatment for MPX, and vaccination against MPX infection is the primary preventive measure. The goal of this study was to use immunoinformatic techniques to design novel multi-epitope MPX vaccine constructs capable of inducing immunogenic responses in infected individuals.

Three outer membrane and extracellular MPXV proteins were retrieved based on parameters such as antigenic behavior, non-allergenic and non-toxic nature, and virulence capabilities, to identify T-cell and B-cell epitopes. This method determines the suitability of vaccine candidates for experimental validation during vaccine development (64). T-cell epitopes (MHC-I and MHC-II) are crucial for adaptive immunity. MHC-I epitopes induce durable immunity to eliminate viruses and infected cells from the host, whereas MHC-II epitopes are responsible for generating both cellular and humoral immune responses (65). These epitopes induce a CD4+ helper T-cell response, leading to CD8+ T-cell memory generation and B-cell activation (66, 67). MHC-I and MHC-II epitopes overlapping B-cell epitopes were selected and linked using multiple combinations of immune enhancers and four different adjuvant peptide sequences to design vaccine constructs. ANTIGENpro and Vaxijen v2.0 determined high antigenicity scores for the designed multi-epitope constructs. All designed vaccine constructs were non-allergens and non-toxins. These immunological properties strengthen its potential as a vaccine candidate. physicochemical properties of the predicted vaccine constructs were also investigated.

Structural information is important for vaccine development to study interactions between antigens and receptor molecules. The 3D structures of the vaccine constructs were predicted and further improved after refinement. The refined 3D structural analysis confirmed the structural stability of the designed vaccine constructs and showed the maximum number of residues in the favorable region of the Ramachandran plot. The high-quality predicted structures of the multi-epitope vaccine constructs were validated using ERRAT and ProSA-web predictions. The molecular weights of the vaccine constructs were within the desired range (> 30 kDa). The physicochemical properties of these constructs revealed that they are highly soluble and extremely stable upon expression. The solubility of an overexpressed recombinant peptide in an *E. coli* expression system is a crucial prerequisite for most functional and biochemical studies (68). Additionally, the estimated instability scores infer that the designed vaccine constructs will be extremely stable upon expression, thus enhancing its potential as a vaccine. Confirmation of immunoreactivity based on serological analysis is a key step in validating a constructed vaccine (69) that must be expressed in a suitable expression system. The *E. coli* expression system is considered the most suitable for the cloning and development of recombinant peptides (70, 71).

Molecular docking analysis was performed to examine the ability of the designed vaccines to bind to the TLR4 immune cell receptor. TLR receptors are important for immune cells activation to generate adaptive immune responses and hence play a significant role in innate immunity. Previous studies have reported the involvement of TLR4 in the recognition of viral peptide structures, which trigger the production of inflammatory cytokines (72, 73). Molecular docking analysis confirmed strong binding affinities between the vaccine constructs and the active site of the receptor protein. This determines the ability of the designed vaccine to generate stable immunogenic responses. The best, stable, and most effective vaccine candidate was selected based on docking poses, interacting atoms, and binding free energies. MPXV-V2 was selected as the best vaccine construct based on its lowest global energy and was considered for immune simulation and molecular dynamic simulation studies.

The predictions of the immune simulation results were similar to those of the natural cellular immunogenic responses. Repeated exposure to antigens induced a strong immune response. The development of memory in B- and T-cells was observed with a long-lasting adaptive immunity as memory in B-cells lasts for several months. High levels of T-cytotoxic, T-helper cells, and Ig production were observed, along with an increase in IFN-γ and IL-2 levels after the first injection. The consistent increase in levels upon repeated antigen exposure indicated humoral responses to the vaccine. Simpson index D for the investigation of clonal specificity suggests the possibility of diverse immunogenic responses. The stability of the lead vaccine (MPXV-V2-TLR4) docked complex was validated using MD simulations. This analysis confirmed the strong molecular interactions of the vaccine with the immune receptor, thus ensuring the molecular stability of the multi-epitope vaccine complex in a cellular environment. This implies that the vaccine construct designed in this study is capable of generating strong immune responses with high gene expression. *In silico* investigations utilizing immunoinformatics, techniques are helpful and can contribute to direct laboratory assays, thereby saving time and money. The next step is *in vitro* immunological assays to validate the designed vaccine, evaluate the immunogenicity of the multi-epitope vaccine construct, and design a challenge-protection preclinical trial to validate the results of this study.

## Limitations

This study highlights a multi-epitope-based vaccine design of the MPXV protein component, which is an alternative approach to tackle antigenic complexity. The current study has some limitations. The design of vaccine constructs based on immunoinformatics relies on many predictions. The accuracy of these prediction methods is not perfect, and the degree of protection against MPXV infection is uncertain. Moreover, the immunoinformatics approach has several challenges, including standard benchmarking, limited prediction approaches, and a lack of exact datasets for various computational analyses. Several successful cases have been reported in recent years (74). Therefore, the prediction results, i.e. the proposed vaccine construct awaits investigation using *in vitro* and *in -vivo* bioassays for experimental validation to prove its safety.

## Conclusion

In the present study, reverse vaccinology and immuno-informatics approaches were used to identify potential therapeutic vaccine candidate proteins against the emerging MPXV, based on antigenicity, allergenicity, toxicity, and virulence. Overlapping B- and T-cell epitopes from the selected proteins were used to design multi-epitope-based vaccine constructs. The population coverage of the selected epitopes was 100% worldwide and were conserved among different strains of MPXV, hence ensuring broader protection against different strains of the virus globally. These epitopes were joined using suitable linker and adjuvant sequences to enhance the immunogenicity of the vaccines. Immunological and physiochemical analysis identified MPXV-V2 as an ideal vaccine construct with the lowest global energy and binding affinity for the TLR4 immune receptor. MPXV-V2 has the capability of effective gene expression in the *E. coli* expression system. Moreover, immune stimulation, revealed that the MPXV-V2 vaccine has the ability to generate humoral and cellular immune responses against the MPXV. The MD simulation analysis confirmed the stability of the vaccine in the cellular environment. Additional experimental and clinical assays are required to validate the results of the present study.

## Data availability statement

The original contributions presented in the study are included in the article/**Supplementary Material**. Further inquiries can be directed to the corresponding authors.

## Author contributions

CL, and SA conceived the basic idea. SA, FA, NR, and AA performed the analysis and prepared the initial draft. QF, YA, LR, AK, and AK reviewed the critical analysis and helped in draft preparation. NR, CL finalized the draft and supervised the overall study. All authors read and approved the final manuscript.

## Funding

This work was supported by the National Natural Science Foundation of China [31971180].

## Conflict of interest

The authors declare that the research was conducted in the absence of any commercial or financial relationships that could be construed as a potential conflict of interest.

## Publisher’s note

All claims expressed in this article are solely those of the authors and do not necessarily represent those of their affiliated organizations, or those of the publisher, the editors and the reviewers. Any product that may be evaluated in this article, or claim that may be made by its manufacturer, is not guaranteed or endorsed by the publisher.

## Abbreviations

MPXV, Monkeypox Virus; CDC, Centers for Disease Control and Prevention; WHO, World Health Organization; NCBI, National Center for Biotechnology Information; BLAST, Basic Local Alignment Search Tool; CTL, Cytotoxic T Lymphocyte; MHC-I, Major Histocompatibility Complex Class I; MHC-II, Major Histocompatibility Complex Class II; IEDB, Immune Epitope Database; SMM, Stabilize Matrix Method; LPS, Lipopolysaccharide; JCAT, Java Codon Adaptation Tool; CAI, Codon Adaptation Index; NMA, Normal Mode Analysis; MD, Molecular Dynamic; RMSD, Root-Mean-Square Deviation.
