# Supplementary material for: Multi-epitope chimeric vaccine design against emerging Monkeypox virus via reverse vaccinology techniques- a bioinformatics and immunoinformatics approach
Source: Front Immunol. 2022 Aug 25;13:985450. doi: 10.3389/fimmu.2022.985450 (PMC9452969; doi:10.3389/fimmu.2022.985450)
Supplement: Supplementary file 3 [file Table_1.docx]

**Table S1:** The shortlisted non-human homologous viral proteins analysis for antigenicity, allergenicity, toxicity and virulence.

| **S/No** | **Protein GenBank-IDs** | **Allergenicity**  **AllerTOP2.0** | **VaxiJen 2.0**  **>0.4 threshold** | **ToxinPred** | **Virulentpred** |
| --- | --- | --- | --- | --- | --- |
| 1 | URP85107 | Probable Non-Allergen | 0.4123  Antigen | Too long | 1.0434  Virulent |
| 2 | URP84945 | Probable Non-Allergen | 0.4018  Antigen | Too long | 1.0391  Virulent |
| 3 | URP84997 | Probable Non-Allergen | 0.4124  Antigen | Too long | 1.0417  Virulent |
| 4. | URP85116 | Probable Non-Allergen | 0.4225  Antigen | Too long | 1.1020  Virulent |
| 5. | URP85008 | Probable Non-Allergen | 0.4193  Antigen | Too long | 1.0773  Virulent |
| 6. | URP85109 | Probable Non-Allergen | 0.5734  Antigen | some toxic  peptides | 1.0173  Virulent |
| 7. | URP84967 | Probable Non-Allergen | 0.4473  Antigen | some toxic  peptides | 1.0753  Virulent |
| 8. | URP85110 | Probable Non-Allergen | 0.4621  Antigen | some toxic  peptides | 0.8288  Virulent |
| 9. | URP84966 | Probable Non-Allergen | 0.5992  Antigen | Non-Toxin | 1.0756  Virulent |
| 10. | URP85030 | Probable Non-Allergen | 0.5054  Antigen | Too long | 0.5484  Virulent |
| 11. | URP84950 | Probable Non-Allergen | 0.5117  Antigen | some toxic  peptides | 0.9939  Virulent |
| 12. | URP85049 | Probable Non-Allergen | 0.5316  Antigen | Non-Toxin | 1.1259  Virulent |
| 13. | URP84978 | Probable Non-Allergen | 0.4754  Antigen | some toxic  peptides | 1.0369  Virulent |
| 14. | URP85065 | Probable Non-Allergen | 0.5482  Antigen | Too Long | 0.6515  Virulent |
| 15. | URP85108 | Probable Non-Allergen | 0.4760  Antigen | Too Long | 1.0322  Virulent |
| 16. | URP85119 | Probable Non-Allergen | 0.4753  Antigen | some toxic  peptides | 0.9842  Virulent |
| 17. | URP85088 | Probable Non-Allergen | 0.4098  Antigen | some toxic  peptides | 1.0041  Virulent |
| 18. | URP85121 | Probable Non-Allergen | 0.6463  Antigen | some toxic  peptides | 1.0931  Virulent |
| 19. | URP85099 | Probable Non-Allergen | 0.5358  Antigen | Non-Toxin | 0.9799  Virulent |
| 20. | URP85090 | Probable Non-Allergen | 0.4324  Antigen | some toxic  peptides | 1.1139  Virulent |
| 21. | URP85128 | Probable Non-Allergen | 0.5320  Antigen | Non-Toxin | -0.445  Non-Virulent |
| 22. | URP84965 | Probable Non-Allergen | 0.5423  Antigen | Non-Toxin | 1.1254  Virulent |
| 23. | URP84960 | Probable Non-Allergen | 0. 4156  Antigen | Non-Toxin | 1.0900  Virulent |
| 24. | URP84992 | Probable Non-Allergen | 0.4188  Antigen | some toxic  peptides | 0.9388  Virulent |

**Table S2:** Designed vaccine constructs with allergeicity, antigenicity and solubility analysis.

| **Vaccine construct** | **Adjuvant** | **Construct** | **Allergenicity** | **Antigenpro** | **Vaxijen** | **Solubility** |
| --- | --- | --- | --- | --- | --- | --- |
| MPXV-V1 | Hbha adjuvant | EAAAKMAENPNIDDLPAPLLAALGAADLALATVNDLIANLRERAEETRAETRTRVEERRARLTKFQEDLPEQFIELRDKFTTEELRKAAEGYLEAATNRYNELVERGEAALQRLRSQTAFEDASARAEGYVDQAVELTQEALGTVASQTRAVGERAAKLVGIELEAAAKAKFVAAWTLKAAAGGGSSNEEFDPVDDGPVSDYVSELYGGGSLPAVVYSTCTVPTMNNAKLTGGGSAKFVAAWTLKAAAGGGSYISCTANSWNVIPSCQQKCDHEYGAEALERAGNKINSIVERRSGMSNVVDSTHEYGAEALERAGAKFVAAWTLKAAAGGGSVAEASTIMVATARSSPEELEGGGSTKTVPMMNVVTKLQGNTITIHEYGAEALERAGVHWNKKKYSSYEEAKKHDDGHEYGAEALERAGAKFVAAWTLKAAAGGGSSSSNHEGKPHYITENYRNPYGGGSVRINFKGGYISGGFLPNEYVHEYGAEALERAGAKFVAAWTLKAAAGGGS | -0.45201802 (ALlgPred-Non Allergen)  Non allergen-Allertop | 0.917568 | 0.4781 | 0.980809 |
| V2 | Beta defensin adjuvant | EAAAKGIINTLQKYYCRVRGGRCAVLSCLPKEEQIGKCSTRGRKCCRRKKEAAAKAKFVAAWTLKAAAGGGSSNEEFDPVDDGPVSDYVSELYGGGSLPAVVYSTCTVPTMNNAKLTGGGSAKFVAAWTLKAAAGGGSYISCTANSWNVIPSCQQKCDHEYGAEALERAGNKINSIVERRSGMSNVVDSTHEYGAEALERAGAKFVAAWTLKAAAGGGSVAEASTIMVATARSSPEELEGGGSTKTVPMMNVVTKLQGNTITIHEYGAEALERAGVHWNKKKYSSYEEAKKHDDGHEYGAEALERAGAKFVAAWTLKAAAGGGSSSSNHEGKPHYITENYRNPYGGGSVRINFKGGYISGGFLPNEYVHEYGAEALERAGAKFVAAWTLKAAAGGGS | -0.21515716 (ALlgPred-Allergen)  Non allergen-Allertop | 0.933022 | 0.4759 | 0.972829 |
| V3 | Hbha cons  rved adjuvant | EAAAKMAENSNIDDIKAPLLAALGAADLALATVNELITNLRERAEETRRSRVEESRARLTKLQEDLPEQLTELREKFTAEELRKAAEGYLEAATSELVERGEAALERLRSQQSFEEVSARAEGYVDQAVELTQEALGTVASQVEGRAAKLVGIELEAAAKAKFVAAWTLKAAAGGGSSNEEFDPVDDGPVSDYVSELYGGGSLPAVVYSTCTVPTMNNAKLTGGGSAKFVAAWTLKAAAGGGSYISCTANSWNVIPSCQQKCDHEYGAEALERAGNKINSIVERRSGMSNVVDSTHEYGAEALERAGAKFVAAWTLKAAAGGGSVAEASTIMVATARSSPEELEGGGSTKTVPMMNVVTKLQGNTITIHEYGAEALERAGVHWNKKKYSSYEEAKKHDDGHEYGAEALERAGAKFVAAWTLKAAAGGGSSSSNHEGKPHYITENYRNPYGGGSVRINFKGGYISGGFLPNEYVHEYGAEALERAGAKFVAAWTLKAAAGGGS | -0.3656548 (ALlgPred-Allergen)  Non allergen-Allertop | 0.908368 | 0.4740 | 0.987348 |
| V4 | Ribosomal protein adjuvant | EAAAKMAKLSTDELLDAFKEMTLLELSDFVKKFEETFEVTAAAPVAVAAAGAAPAGAAVEAAEEQSEFDVILEAAGDKKIGVIKVVREIVSGLGLKEAKDLVDGAPKPLLEKVAKEAADEAKAKLEAAGATVTVKEAAAKAKFVAAWTLKAAAGGGSSNEEFDPVDDGPVSDYVSELYGGGSLPAVVYSTCTVPTMNNAKLTGGGSAKFVAAWTLKAAAGGGSYISCTANSWNVIPSCQQKCDHEYGAEALERAGNKINSIVERRSGMSNVVDSTHEYGAEALERAGAKFVAAWTLKAAAGGGSVAEASTIMVATARSSPEELEGGGSTKTVPMMNVVTKLQGNTITIHEYGAEALERAGVHWNKKKYSSYEEAKKHDDGHEYGAEALERAGAKFVAAWTLKAAAGGGSSSSNHEGKPHYITENYRNPYGGGSVRINFKGGYISGGFLPNEYVHEYGAEALERAGAKFVAAWTLKAAAGGGS | 0.3866291 (ALlgPred-Allergen)  Non allergen-Allertop | 0.919179 | 0.4489 | 0.993725 |

**Table S3:** Codon optimization of vaccine constructs using JCAT resource.

| **Vaccine construct** | **Protein Sequence** | **Codon Sequence (default)** |
| --- | --- | --- |
| **Hbha adjuvant**  **MPXV-V1** | EAAAKMAENPNIDDLPAPLLAALGAADLALATVNDLIANLRERAEETRAETRTRVEERRARLTKFQEDLPEQFIELRDKFTTEELRKAAEGYLEAATNRYNELVERGEAALQRLRSQTAFEDASARAEGYVDQAVELTQEALGTVASQTRAVGERAAKLVGIELEAAAKAKFVAAWTLKAAAGGGSSNEEFDPVDDGPVSDYVSELYGGGSLPAVVYSTCTVPTMNNAKLTGGGSAKFVAAWTLKAAAGGGSYISCTANSWNVIPSCQQKCDHEYGAEALERAGNKINSIVERRSGMSNVVDSTHEYGAEALERAGAKFVAAWTLKAAAGGGSVAEASTIMVATARSSPEELEGGGSTKTVPMMNVVTKLQGNTITIHEYGAEALERAGVHWNKKKYSSYEEAKKHDDGHEYGAEALERAGAKFVAAWTLKAAAGGGSSSSNHEGKPHYITENYRNPYGGGSVRINFKGGYISGGFLPNEYVHEYGAEALERAGAKFVAAWTLKAAAGGGS | GAGGCCGCCGCCAAGATGGCCGAGAACCCCAACATCGACGACCTGCCCGCCCCCCTGCTGGCCGCCCTGGGCGCCGCCGACCTGGCCCTGGCCACCGTGAACGACCTGATCGCCAACCTGCGCGAGCGCGCCGAGGAGACCCGCGCCGAGACCCGCACCCGCGTGGAGGAGCGCCGCGCCCGCCTGACCAAGTTCCAGGAGGACCTGCCCGAGCAGTTCATCGAGCTGCGCGACAAGTTCACCACCGAGGAGCTGCGCAAGGCCGCCGAGGGCTACCTGGAGGCCGCCACCAACCGCTACAACGAGCTGGTGGAGCGCGGCGAGGCCGCCCTGCAGCGCCTGCGCAGCCAGACCGCCTTCGAGGACGCCAGCGCCCGCGCCGAGGGCTACGTGGACCAGGCCGTGGAGCTGACCCAGGAGGCCCTGGGCACCGTGGCCAGCCAGACCCGCGCCGTGGGCGAGCGCGCCGCCAAGCTGGTGGGCATCGAGCTGGAGGCCGCCGCCAAGGCCAAGTTCGTGGCCGCCTGGACCCTGAAGGCCGCCGCCGGCGGCGGCAGCAGCAACGAGGAGTTCGACCCCGTGGACGACGGCCCCGTGAGCGACTACGTGAGCGAGCTGTACGGCGGCGGCAGCCTGCCCGCCGTGGTGTACAGCACCTGCACCGTGCCCACCATGAACAACGCCAAGCTGACCGGCGGCGGCAGCGCCAAGTTCGTGGCCGCCTGGACCCTGAAGGCCGCCGCCGGCGGCGGCAGCTACATCAGCTGCACCGCCAACAGCTGGAACGTGATCCCCAGCTGCCAGCAGAAGTGCGACCACGAGTACGGCGCCGAGGCCCTGGAGCGCGCCGGCAACAAGATCAACAGCATCGTGGAGCGCCGCAGCGGCATGAGCAACGTGGTGGACAGCACCCACGAGTACGGCGCCGAGGCCCTGGAGCGCGCCGGCGCCAAGTTCGTGGCCGCCTGGACCCTGAAGGCCGCCGCCGGCGGCGGCAGCGTGGCCGAGGCCAGCACCATCATGGTGGCCACCGCCCGCAGCAGCCCCGAGGAGCTGGAGGGCGGCGGCAGCACCAAGACCGTGCCCATGATGAACGTGGTGACCAAGCTGCAGGGCAACACCATCACCATCCACGAGTACGGCGCCGAGGCCCTGGAGCGCGCCGGCGTGCACTGGAACAAGAAGAAGTACAGCAGCTACGAGGAGGCCAAGAAGCACGACGACGGCCACGAGTACGGCGCCGAGGCCCTGGAGCGCGCCGGCGCCAAGTTCGTGGCCGCCTGGACCCTGAAGGCCGCCGCCGGCGGCGGCAGCAGCAGCAGCAACCACGAGGGCAAGCCCCACTACATCACCGAGAACTACCGCAACCCCTACGGCGGCGGCAGCGTGCGCATCAACTTCAAGGGCGGCTACATCAGCGGCGGCTTCCTGCCCAACGAGTACGTGCACGAGTACGGCGCCGAGGCCCTGGAGCGCGCCGGCGCCAAGTTCGTGGCCGCCTGGACCCTGAAGGCCGCCGCCGGCGGCGGCAGC |
| **Beta definsin adjuvant**  **MPXV-V2** | EAAAKGIINTLQKYYCRVRGGRCAVLSCLPKEEQIGKCSTRGRKCCRRKKEAAAKAKFVAAWTLKAAAGGGSSNEEFDPVDDGPVSDYVSELYGGGSLPAVVYSTCTVPTMNNAKLTGGGSAKFVAAWTLKAAAGGGSYISCTANSWNVIPSCQQKCDHEYGAEALERAGNKINSIVERRSGMSNVVDSTHEYGAEALERAGAKFVAAWTLKAAAGGGSVAEASTIMVATARSSPEELEGGGSTKTVPMMNVVTKLQGNTITIHEYGAEALERAGVHWNKKKYSSYEEAKKHDDGHEYGAEALERAGAKFVAAWTLKAAAGGGSSSSNHEGKPHYITENYRNPYGGGSVRINFKGGYISGGFLPNEYVHEYGAEALERAGAKFVAAWTLKAAAGGGS | GAGGCCGCCGCCAAGGGCATCATCAACACCCTGCAGAAGTACTACTGCCGCGTGCGCGGCGGCCGCTGCGCCGTGCTGAGCTGCCTGCCCAAGGAGGAGCAGATCGGCAAGTGCAGCACCCGCGGCCGCAAGTGCTGCCGCCGCAAGAAGGAGGCCGCCGCCAAGGCCAAGTTCGTGGCCGCCTGGACCCTGAAGGCCGCCGCCGGCGGCGGCAGCAGCAACGAGGAGTTCGACCCCGTGGACGACGGCCCCGTGAGCGACTACGTGAGCGAGCTGTACGGCGGCGGCAGCCTGCCCGCCGTGGTGTACAGCACCTGCACCGTGCCCACCATGAACAACGCCAAGCTGACCGGCGGCGGCAGCGCCAAGTTCGTGGCCGCCTGGACCCTGAAGGCCGCCGCCGGCGGCGGCAGCTACATCAGCTGCACCGCCAACAGCTGGAACGTGATCCCCAGCTGCCAGCAGAAGTGCGACCACGAGTACGGCGCCGAGGCCCTGGAGCGCGCCGGCAACAAGATCAACAGCATCGTGGAGCGCCGCAGCGGCATGAGCAACGTGGTGGACAGCACCCACGAGTACGGCGCCGAGGCCCTGGAGCGCGCCGGCGCCAAGTTCGTGGCCGCCTGGACCCTGAAGGCCGCCGCCGGCGGCGGCAGCGTGGCCGAGGCCAGCACCATCATGGTGGCCACCGCCCGCAGCAGCCCCGAGGAGCTGGAGGGCGGCGGCAGCACCAAGACCGTGCCCATGATGAACGTGGTGACCAAGCTGCAGGGCAACACCATCACCATCCACGAGTACGGCGCCGAGGCCCTGGAGCGCGCCGGCGTGCACTGGAACAAGAAGAAGTACAGCAGCTACGAGGAGGCCAAGAAGCACGACGACGGCCACGAGTACGGCGCCGAGGCCCTGGAGCGCGCCGGCGCCAAGTTCGTGGCCGCCTGGACCCTGAAGGCCGCCGCCGGCGGCGGCAGCAGCAGCAGCAACCACGAGGGCAAGCCCCACTACATCACCGAGAACTACCGCAACCCCTACGGCGGCGGCAGCGTGCGCATCAACTTCAAGGGCGGCTACATCAGCGGCGGCTTCCTGCCCAACGAGTACGTGCACGAGTACGGCGCCGAGGCCCTGGAGCGCGCCGGCGCCAAGTTCGTGGCCGCCTGGACCCTGAAGGCCGCCGCCGGCGGCGGCAGC |
| **Hbha conserved adjuvant**  **MPXV-V3** | EAAAKMAENSNIDDIKAPLLAALGAADLALATVNELITNLRERAEETRRSRVEESRARLTKLQEDLPEQLTELREKFTAEELRKAAEGYLEAATSELVERGEAALERLRSQQSFEEVSARAEGYVDQAVELTQEALGTVASQVEGRAAKLVGIELEAAAKAKFVAAWTLKAAAGGGSSNEEFDPVDDGPVSDYVSELYGGGSLPAVVYSTCTVPTMNNAKLTGGGSAKFVAAWTLKAAAGGGSYISCTANSWNVIPSCQQKCDHEYGAEALERAGNKINSIVERRSGMSNVVDSTHEYGAEALERAGAKFVAAWTLKAAAGGGSVAEASTIMVATARSSPEELEGGGSTKTVPMMNVVTKLQGNTITIHEYGAEALERAGVHWNKKKYSSYEEAKKHDDGHEYGAEALERAGAKFVAAWTLKAAAGGGSSSSNHEGKPHYITENYRNPYGGGSVRINFKGGYISGGFLPNEYVHEYGAEALERAGAKFVAAWTLKAAAGGGS | GAGGCCGCCGCCAAGATGGCCGAGAACAGCAACATCGACGACATCAAGGCCCCCCTGCTGGCCGCCCTGGGCGCCGCCGACCTGGCCCTGGCCACCGTGAACGAGCTGATCACCAACCTGCGCGAGCGCGCCGAGGAGACCCGCCGCAGCCGCGTGGAGGAGAGCCGCGCCCGCCTGACCAAGCTGCAGGAGGACCTGCCCGAGCAGCTGACCGAGCTGCGCGAGAAGTTCACCGCCGAGGAGCTGCGCAAGGCCGCCGAGGGCTACCTGGAGGCCGCCACCAGCGAGCTGGTGGAGCGCGGCGAGGCCGCCCTGGAGCGCCTGCGCAGCCAGCAGAGCTTCGAGGAGGTGAGCGCCCGCGCCGAGGGCTACGTGGACCAGGCCGTGGAGCTGACCCAGGAGGCCCTGGGCACCGTGGCCAGCCAGGTGGAGGGCCGCGCCGCCAAGCTGGTGGGCATCGAGCTGGAGGCCGCCGCCAAGGCCAAGTTCGTGGCCGCCTGGACCCTGAAGGCCGCCGCCGGCGGCGGCAGCAGCAACGAGGAGTTCGACCCCGTGGACGACGGCCCCGTGAGCGACTACGTGAGCGAGCTGTACGGCGGCGGCAGCCTGCCCGCCGTGGTGTACAGCACCTGCACCGTGCCCACCATGAACAACGCCAAGCTGACCGGCGGCGGCAGCGCCAAGTTCGTGGCCGCCTGGACCCTGAAGGCCGCCGCCGGCGGCGGCAGCTACATCAGCTGCACCGCCAACAGCTGGAACGTGATCCCCAGCTGCCAGCAGAAGTGCGACCACGAGTACGGCGCCGAGGCCCTGGAGCGCGCCGGCAACAAGATCAACAGCATCGTGGAGCGCCGCAGCGGCATGAGCAACGTGGTGGACAGCACCCACGAGTACGGCGCCGAGGCCCTGGAGCGCGCCGGCGCCAAGTTCGTGGCCGCCTGGACCCTGAAGGCCGCCGCCGGCGGCGGCAGCGTGGCCGAGGCCAGCACCATCATGGTGGCCACCGCCCGCAGCAGCCCCGAGGAGCTGGAGGGCGGCGGCAGCACCAAGACCGTGCCCATGATGAACGTGGTGACCAAGCTGCAGGGCAACACCATCACCATCCACGAGTACGGCGCCGAGGCCCTGGAGCGCGCCGGCGTGCACTGGAACAAGAAGAAGTACAGCAGCTACGAGGAGGCCAAGAAGCACGACGACGGCCACGAGTACGGCGCCGAGGCCCTGGAGCGCGCCGGCGCCAAGTTCGTGGCCGCCTGGACCCTGAAGGCCGCCGCCGGCGGCGGCAGCAGCAGCAGCAACCACGAGGGCAAGCCCCACTACATCACCGAGAACTACCGCAACCCCTACGGCGGCGGCAGCGTGCGCATCAACTTCAAGGGCGGCTACATCAGCGGCGGCTTCCTGCCCAACGAGTACGTGCACGAGTACGGCGCCGAGGCCCTGGAGCGCGCCGGCGCCAAGTTCGTGGCCGCCTGGACCCTGAAGGCCGCCGCCGGCGGCGGCAGC |
| **Ribosomal protein adjuvant**  **MPXV-V4** | EAAAKMAKLSTDELLDAFKEMTLLELSDFVKKFEETFEVTAAAPVAVAAAGAAPAGAAVEAAEEQSEFDVILEAAGDKKIGVIKVVREIVSGLGLKEAKDLVDGAPKPLLEKVAKEAADEAKAKLEAAGATVTVKEAAAKAKFVAAWTLKAAAGGGSSNEEFDPVDDGPVSDYVSELYGGGSLPAVVYSTCTVPTMNNAKLTGGGSAKFVAAWTLKAAAGGGSYISCTANSWNVIPSCQQKCDHEYGAEALERAGNKINSIVERRSGMSNVVDSTHEYGAEALERAGAKFVAAWTLKAAAGGGSVAEASTIMVATARSSPEELEGGGSTKTVPMMNVVTKLQGNTITIHEYGAEALERAGVHWNKKKYSSYEEAKKHDDGHEYGAEALERAGAKFVAAWTLKAAAGGGSSSSNHEGKPHYITENYRNPYGGGSVRINFKGGYISGGFLPNEYVHEYGAEALERAGAKFVAAWTLKAAAGGGS | GAGGCCGCCGCCAAGATGGCCAAGCTGAGCACCGACGAGCTGCTGGACGCCTTCAAGGAGATGACCCTGCTGGAGCTGAGCGACTTCGTGAAGAAGTTCGAGGAGACCTTCGAGGTGACCGCCGCCGCCCCCGTGGCCGTGGCCGCCGCCGGCGCCGCCCCCGCCGGCGCCGCCGTGGAGGCCGCCGAGGAGCAGAGCGAGTTCGACGTGATCCTGGAGGCCGCCGGCGACAAGAAGATCGGCGTGATCAAGGTGGTGCGCGAGATCGTGAGCGGCCTGGGCCTGAAGGAGGCCAAGGACCTGGTGGACGGCGCCCCCAAGCCCCTGCTGGAGAAGGTGGCCAAGGAGGCCGCCGACGAGGCCAAGGCCAAGCTGGAGGCCGCCGGCGCCACCGTGACCGTGAAGGAGGCCGCCGCCAAGGCCAAGTTCGTGGCCGCCTGGACCCTGAAGGCCGCCGCCGGCGGCGGCAGCAGCAACGAGGAGTTCGACCCCGTGGACGACGGCCCCGTGAGCGACTACGTGAGCGAGCTGTACGGCGGCGGCAGCCTGCCCGCCGTGGTGTACAGCACCTGCACCGTGCCCACCATGAACAACGCCAAGCTGACCGGCGGCGGCAGCGCCAAGTTCGTGGCCGCCTGGACCCTGAAGGCCGCCGCCGGCGGCGGCAGCTACATCAGCTGCACCGCCAACAGCTGGAACGTGATCCCCAGCTGCCAGCAGAAGTGCGACCACGAGTACGGCGCCGAGGCCCTGGAGCGCGCCGGCAACAAGATCAACAGCATCGTGGAGCGCCGCAGCGGCATGAGCAACGTGGTGGACAGCACCCACGAGTACGGCGCCGAGGCCCTGGAGCGCGCCGGCGCCAAGTTCGTGGCCGCCTGGACCCTGAAGGCCGCCGCCGGCGGCGGCAGCGTGGCCGAGGCCAGCACCATCATGGTGGCCACCGCCCGCAGCAGCCCCGAGGAGCTGGAGGGCGGCGGCAGCACCAAGACCGTGCCCATGATGAACGTGGTGACCAAGCTGCAGGGCAACACCATCACCATCCACGAGTACGGCGCCGAGGCCCTGGAGCGCGCCGGCGTGCACTGGAACAAGAAGAAGTACAGCAGCTACGAGGAGGCCAAGAAGCACGACGACGGCCACGAGTACGGCGCCGAGGCCCTGGAGCGCGCCGGCGCCAAGTTCGTGGCCGCCTGGACCCTGAAGGCCGCCGCCGGCGGCGGCAGCAGCAGCAGCAACCACGAGGGCAAGCCCCACTACATCACCGAGAACTACCGCAACCCCTACGGCGGCGGCAGCGTGCGCATCAACTTCAAGGGCGGCTACATCAGCGGCGGCTTCCTGCCCAACGAGTACGTGCACGAGTACGGCGCCGAGGCCCTGGAGCGCGCCGGCGCCAAGTTCGTGGCCGCCTGGACCCTGAAGGCCGCCGCCGGCGGCGGCAGC |
